# 巨大原发性肺淋巴瘤1例并文献复习

**DOI:** 10.3779/j.issn.1009-3419.2010.05.33

**Published:** 2010-05-20

**Authors:** 红荣 郭

**Affiliations:** 213003 常州，南京医科大学附属常州第二人民医院呼吸科 Department of Respiratory Medicine, Afliated to Changzhou No.2 People's Hospital, Nanjing Medical University, Changzhou 213003, China

淋巴瘤在肺部的表现分为原发性和继发性两种。原发性肺淋巴瘤（primary pulmonary lymphoma, PPL）临床少见，其发病率约占恶性淋巴瘤的1%左右。现将我科经病理证实的巨大原发性肺淋巴瘤1例报道如下。

## 1 临床资料

患者，男，73岁，因“咯血伴胸闷、右侧胸痛3天”于2009年5月11日入院，病程中无发热、盗汗，体重无明显减轻。1999年10月因“喉癌”手术治疗（具体诊治经过不详）。患者否认“糖尿病、肺结核”病史。入院时查体：T 37.1 ℃，神志清楚，精神可。呼吸平稳，全身浅表淋巴结无肿大，气管居中。颈前见一手术疤痕。胸廓无畸形，右侧触觉语颤减弱，右肺叩诊浊音，右下肺呼吸音低，两肺未闻及干、湿性啰音。心率78次/分，律齐，各瓣膜听诊区未闻及病理性杂音。腹部平软，无压痛及反跳痛，肝脾未及肿大。双下肢无水肿。神经系统检查阴性。门诊肺CT提示“双肺纹理增多，右下肺见一8 cm×6 cm×6.2 cm巨大软组织肿块影，肿块内见液化坏死，边缘见毛刺样改变，右侧胸前见新月样低密度影，纵隔见肿大淋巴结，右上肺见斑片状模糊影”（图 1A）。入院初步诊断为“右下肺癌伴胸膜腔及纵隔淋巴结转移”。入院后辅助检查：血常规：白细胞7.90×10^9^/L，中性粒细胞80.2%，血红蛋白109 g/L，血沉46 mm/h；PPD试验（-）；风湿全套检测（-）；肝肾功能正常；癌胚抗原（CEA）、非小细胞肺癌抗原（CY-211）、神经特异性烯醇酶（NSE）、甲胎蛋白（AFP）、鳞状上皮癌抗原（SCC）检查均在正常范围。腹部B超显示肝脾肾未见异常，后腹膜未见肿大淋巴结。胸腔穿刺胸水送检提示为渗出液，胸水中未找到癌细胞。纤支镜检查示右中叶支气管粘膜充血，皱襞增粗，表面凹凸不平，内侧段见新生物，外侧段外压性闭塞。新生物活检病理示支气管粘膜间质中见弥漫小细胞浸润，免疫组化示：CK（-），CK7（-），LCA（+++），CD20（+++），CD3（+），提示恶性B细胞性淋巴瘤（图 2，图 3）。因此诊断为“原发性肺淋巴瘤（非霍杰金淋巴瘤）”。由于患者高龄且伴胸膜腔、纵隔淋巴结受累，明确诊断后未予常规CHOP方案（环磷酰胺、阿霉素、长春新碱、泼尼松）治疗，而给予FC方案（氟达拉滨+环磷酰胺）化疗，共3个疗程，症状明显改善，胸部CT提示肺部肿块明显缩小，胸水控制良好（图 1B，图 1C）。目前仍在随访中。

**1:**
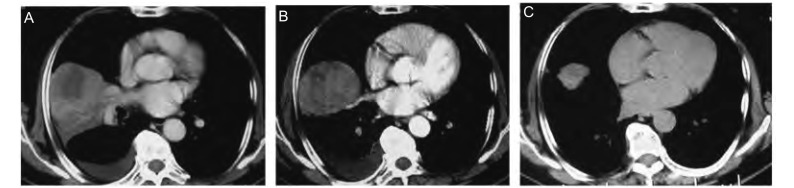
胸部CT显示右肺下叶肿块的改变。A：CT显示右下肺见一范围约8 cm×6 cm×6.2 cm的软组织肿块影（2009-05-08）；B：第一次化疗后肿块较前缩小，胸水减少（2009-06-10）；C：第三次化疗后肿块较前明显缩小，胸水消失（2009-08-11） The changes of chest CT scan. A: Chest CT scan shows a 8 cm×6 cm×6.2 cm lobulated mass in the lower right lobe (2009-05-08); B: The mass is become smaller after the first chemotherapy (2009-06-10); C: The mass is become smaller obviously and pleural effusions disappear after the third chemotherapy (2009-08-11)

**2:**
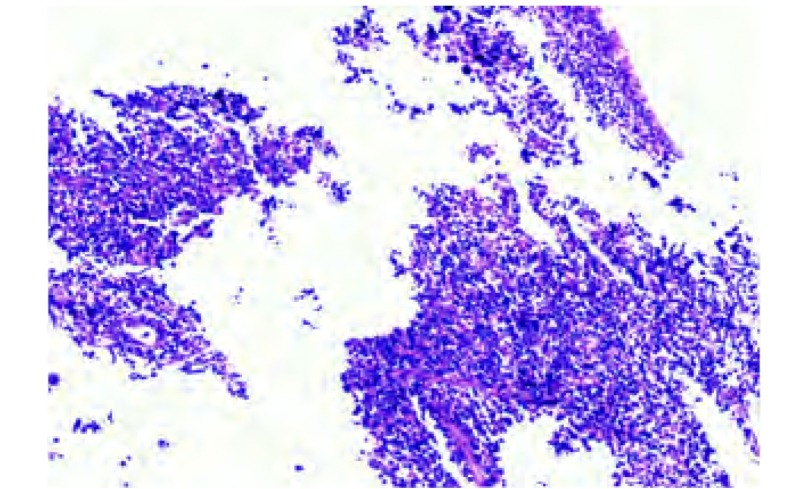
支气管粘膜中见弥散小细胞浸润（HE, ×150） The small cells were seen in the bronchial mucous membrane (HE, ×150)

**3:**
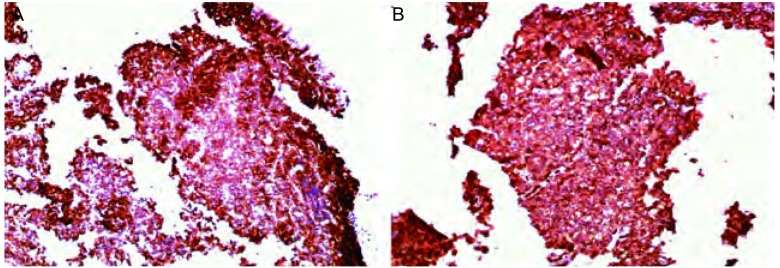
免疫组化CD20（+++）（A）和LCA（+++）（B）（IHC, ×300） Immunohistochemistry for CD20 (A) and LCA (B) were obviously positive (IHC, ×300)

## 2 讨论

PPL大多起源于支气管粘膜相关的淋巴组织^[1]^，病理学上分为非霍杰金淋巴瘤和霍杰金淋巴瘤两大类，绝大多数为非霍杰金淋巴瘤，而且多数为粘膜相关淋巴组织淋巴瘤，以B细胞淋巴瘤为主。Kim等^[2]^提出本病的诊断标准为：①有明确的组织病理学依据；②病变累及单侧或双侧肺，伴或不伴肺门、纵隔淋巴结受侵；③无其它淋巴结或结外组织器官受侵；④排除纵隔腺瘤；⑤无淋巴瘤病史；⑥确诊后3个月内无胸外器官受侵。

原发性肺淋巴瘤的瘤细胞仅侵犯肺组织的结外淋巴组织，并无淋巴结病变，因而有别于肺门、纵隔淋巴瘤和其它脏器淋巴瘤的肺侵犯。该病男女发病率相似，发病年龄40岁-70岁，平均55岁，与吸烟无关^[3, 4]^。临床表现与淋巴瘤类型有关。Cordier等^[4]^报道的70例病例中，61例为低度恶性淋巴瘤，约半数无症状，其余病例有咳嗽、呼吸困难、胸痛、咯血、伴冷球蛋白血症性血管炎症状；9例高度恶性的病例有消瘦、发热等全身症状。本例患者有咯血、胸闷、胸痛等症状，且有胸膜浸润、胸腔积液、纵隔淋巴结肿大，提示低度恶性淋巴瘤也可进展浸润。

原发性肺淋巴瘤的影像学表现多种多样，有单发或多发结节、软组织肿块影、毛玻璃影、纤维条索影及实变影等，且大多数患者有两种以上的影像学表现。宋伟等^[5]^将其归纳为4种：结节肿块型、肺炎或肺泡型、间质型和粟粒型。本例患者属结节肿块型，且为单发，伴有胸膜腔及纵隔淋巴结转移，与Cordier等^[4]^提出的诊断标准不一致，但符合Kim等^[2]^提出的诊断标准。正是由于影像学的多样性，从影像学角度看原发性肺淋巴瘤无明显特征性改变，容易被误诊为肺癌、肺炎、肺结核、支气管肺泡细胞癌或肿瘤肺淋巴管转移等疾病。因此，肺癌、肺结核、支气管肺泡细胞癌或肿瘤肺淋巴管转移等疾病诊断时应注意鉴别。由于影像改变无特异性，影像诊断考虑该病时应及时取得病理标本，获得明确的病理诊断。原发性肺淋巴瘤的确诊依赖组织病理学检查，开胸肺活检、经皮肺穿刺、纤维支气管镜均可获取组织。结合免疫组化则可使其分型更细，诊断更精确。纤维支气管镜检查一般无阳性发现，目前经皮肺穿刺活检为常用的、创伤性较小而患者易于接受的内科诊断方法。本例患者是通过纤支镜检查确诊，且发现管腔内有新生物。

目前对于原发性肺淋巴瘤的治疗有一定的争论，大多数学者^[6, 7]^认为手术是治疗原发性肺淋巴瘤的主要方法，尤其是一些局限性的病例。手术既有明确诊断的作用，又能达到治疗的目的，同时也为下一步放、化疗提供依据。Frederic等^[7]^认为对于原发性肺淋巴瘤，完整手术切除后患者的10年生存率可达到87.5%，他们的生存期要高于部分手术切除的患者。但对于双侧病变、肺外浸润、复发的病例则应考虑放、化疗。如病理类型为霍杰金淋巴瘤则多采用ABVD（阿霉素+博来霉素+长春新碱+达卡巴嗪）方案、MOPP（氮芥+长春新碱+甲基苄肼+泼尼松）方案或ABVD与MOPP方案交替。如病理类型为非霍杰金淋巴瘤则常用CHOP方案（环磷酰胺+阿霉素+长春新碱+泼尼松）。本例患者高龄，且有胸膜腔、纵隔淋巴结转移，故选用FC方案（氟达拉滨+环磷酰胺）化疗，共3个疗程，症状明显改善，胸部CT提示肺部肿块明显缩小，胸水完全控制。因此，当常规方案化疗效果不理想或患者不能耐受时，FC方案不失为一种较好的化疗方案。

综上，原发性肺淋巴瘤的发病率低，是一种罕见的肺部恶性肿瘤，临床症状以咳嗽、胸闷、咯血等呼吸道症状较常见，影像学表现多种多样，确诊依赖于组织病理学结果，结合免疫组化则更精确。目前治疗以手术为主，术后辅以放疗或化疗。不能耐受手术或无手术指征者则予化疗或放疗。原发性肺淋巴瘤的预后与病理类型有关，低度恶性者病灶多局限于肺内，较少发生转移，预后较好；高度恶性者，因多发生于免疫低下者，预后较差。
